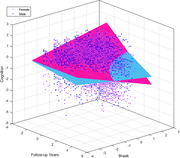# The Role of Sex in Post‐Mortem Neuropathology and Cognitive Decline

**DOI:** 10.1002/alz.095301

**Published:** 2025-01-09

**Authors:** Rowan Dowd, Farooq Kamal, Mahsa Dadar, Michael Oliver, Cassandra Morrison

**Affiliations:** ^1^ Carleton University, Ottawa, ON Canada; ^2^ Douglas Mental Health University Institute, McGill University, Montreal, QC Canada; ^3^ Douglas Mental Health University Institute, Montreal, QC Canada; ^4^ McGill University, Montreal, QC Canada; ^5^ Belmont University, Nashville, TN USA

## Abstract

**Background:**

Females tend to exhibit more Alzheimer’s disease (AD) pathology and have more clinical symptoms compared to males with similar levels of pathology. Post‐mortem studies in patients with AD have revealed that neuropathology is related to cognitive decline trajectories before death. However, what remains unclear is the intersection between sex differences in post‐mortem pathology and cognitive decline. These sex differences remain unstudied and may provide important treatment options in reducing pathology and delaying cognitive decline.

**Method:**

1978 participants (females = 1354) from the RADC RUSH dataset with diagnoses at death including cognitively normal, mild cognitive impairment, and dementia were included. Pathological variables included neurofibrillary tangles (NFT), tangles, Braak staging, amyloid, and hippocampal sclerosis. Linear mixed models examined the relationship between pathology at death and rate of global cognitive decline before death between males and females.

**Result:**

At death, females exhibited higher NFT burden (*p*<.001), tangles (*p*<.001), and Braak staging (*p*<.001) compared to males. Neither amyloid nor hippocampal sclerosis differed between males and females. Both females and males rate of global cognitive change was negatively associated with NFT burden (*p*<.001), tangles (*p*<.001), Braak staging (*p*<.001), amyloid burden (*p*<.001), with higher levels of pathology associating with worse cognitive decline. Only females exhibited a negative association between hippocampal sclerosis and worse cognition (*p*<.001). Importantly, the relationships between pathology and cognition were stronger for females than males for all pathology measures (see Fig. 1 for Braak example). To ensure the differences in rate of cognitive change associated with pathology between males and females were not due to differences in age or amount of pathology, a secondary analysis was completed on a subsample of males and females matched based on age and level of pathology. Results obtained were similar in terms of significance and effect size indicating that females are more negatively affected by AD‐related pathology.

**Conclusion:**

Post‐mortem pathology is strongly associated with cognitive decline before death, with the relationship being stronger in females than males. To mitigate cognitive decline and alleviate stress on the healthcare system, targeting females earlier in the lifespan with interventions such as lifestyle modification reduce disparities in sex differences in AD incidence.